# Editorial Introduction

**DOI:** 10.1089/heq.2022.0092

**Published:** 2022-06-02

**Authors:** Zhuo Chen, Grace Ma, Monica McLemore

**Affiliations:** ^1^ Department of Health Policy and Management, College of Public Health, University of Georgia, Athens, Georgia, USA.; ^2^ Department of Urban Health & Population Science, Lewis Katz School of Medicine, Temple University Health Sciences Center, Philadelphia, Pennsylvania, USA.; ^3^ Department of Family Health Care Nursing, School of Nursing, University of California San Francisco, San Francisco, California, USA.

May 14, 2022, was a sad day, another sad day. Ten people were killed in a senseless racist motivated shooting.^1^ Eleven of the 13 people shot were Black.

Three days before the day, May 11, three Asian women were wounded in a series of shootings at Asian American businesses.^2^

About 14 months ago, eight died in a spree of shootings at three locations in Atlanta, six of whom were Asian women.^3^

Those are only three of a long, unfortunately growing, list of hate crimes against Asian Americans and Black Americans. A small group of extremists do not believe in “all men are created equal” but instead subscribe to the false creed of “all white men are created equal, those with dark or yellowish complexion are different.” Systemic efforts are needed to stop hate crimes against all racial and ethnic groups.

Asian American, Native Hawaiian, and Pacific Islander (AANHPI), who are often stereotyped as “forever foreigners” because of their appearance and culture, have suffered from systemic racism starting from the “Chinese Exclusion Act” in 1882, the Japanese Internment Camps during 1942 and 1945, and to this day the anti-Asian hate crimes that have been exacerbated during the COVID-19 pandemic.^4^ The injustices have profound consequences on AANHPI health.

As we mourn the lives lost in Buffalo and Atlanta, we introduce the special issue on AANHPI health as a somber celebration of the AANHPI Heritage Month.^5^

This special issue is a collection of short reports, research articles, perspectives, and editorials on AANHPI health disparities. The articles touch on the challenges to AANHPI health, the underlying factors, and the diversity within the AANHPI. In an invited commentary, Ka'ai et al. from the White House Initiative on AANHPIs discussed the urgency of addressing health disparities experienced by the AANHPI communities and highlighted key policy priorities, including anti-Asian hate and violence, data disaggregation, and language access.^6^

The special issue was conceptualized during a conversation between Dr. Xinzhi Zhang, the former acting editor-in-chief, and Dr. Zhuo Chen, associate editor of the journal. Dr. Grace Ma, co-guest editor of the special issue, and Dr. Monica McLemore, editor-in-chief, quickly lent their support. We appreciate the support from the China Medical Board and the California Blue Cross Foundation in promoting and disseminating research on AANHPI health.

The collection will continue to grow as we celebrate the AANHPI heritage month and the accomplishments of AANHPI communities. Our thoughts are with the families of the victims of the Buffalo shooting, the Atlanta spa shooting, and all the hate crimes that are against American values and should not have happened. We must be united to condemn and combat racism, xenophobia, and intolerance against AANHPIs in the United States.

## Abbreviations Used

AANHPI: Asian American, Native Hawaiian, and Pacific Islander

## References

[1] Michel L, Tsujimoto B, Becker M. The Buffalo News. May 14, 2022. Available at https://buffalonews.com/news/local/crime-and-courts/buffalos-worst-mass-shooting-takes-10-lives-leaves-3-wounded-attack-called-a-racially-motivated/article_6e8132fa-d3b7-11ec-a714-2b3fbeaf848c.html Accessed May 15, 2022.

[2] Rina Torchinsky. NPR News. May 14, 2022. Available at https://www.npr.org/2022/05/14/1098944521/dallas-asian-korean-salon-shooting-hate-crime Accessed May 22, 2022.

[3] The New York Times. Published March 17, 2021, updated March 26, 2021. Available at https://www.nytimes.com/live/2021/03/17/us/shooting-atlanta-acworth Accessed May 15, 2022.

[4] Shi L, Zhang D, Martin E, et al. Racial discrimination, mental health and behavioral health during the COVID-19 pandemic: a national survey in the United States. J Gen Intern Med. 2022. Apr 11:1–9. doi: 10.1007/s11606-022-07540-2. Epub ahead of print. PMID: ; PMCID: 35411530 PMC8999987

[5] The White House. April 30, 2021. Available at https://www.whitehouse.gov/briefing-room/presidential-actions/2021/04/30/a-proclamation-on-asian-american-and-native-hawaiian-pacific-islander-heritage-month-2021/ Accessed May 15, 2022.

[6] Ka'ai K, Lee R, Chau V, et al. 2022. Advancing Health Equity for Asian Americans, Native Hawaiians, and Pacific Islanders. Health Equity. 2022;6:399–401. 10.1089/heq.2022.0075 PMC9257544 35801149

